# Semaphorin-3F/Neuropilin-2 Transcriptional Expression as a Predictive Biomarker of Occult Lymph Node Metastases in HNSCC

**DOI:** 10.3390/cancers14092259

**Published:** 2022-04-30

**Authors:** Carla Meler-Claramonte, Francesc Xavier Avilés-Jurado, Isabel Vilaseca, Ximena Terra, Paloma Bragado, Gemma Fuster, Xavier León Vintró, Mercedes Camacho

**Affiliations:** 1 Doctoral Programme in Biomedicine, Universitat Rovira i Virgili, 43002 Tarragona, Spain; 2 Otorhinolaryngology Department, Hospital Universitari Joan XXIII de Tarragona, 43005 Tarragona, Spain; 3 IISPV, Institut d’Investigació Sanitària Pere Virgili, 43002 Tarragona, Spain; faviles@clinic.cat; 4 Head Neck Section, Otorhinolaryngology Department, Hospital Universitari Joan XXIII de Tarragona, 43005 Tarragona, Spain; 5 Medicine and Surgery Department, Faculty of Medicine, Universitat Rovira i Virgili, 43002 Tarragona, Spain; 6 Agència de Gestió d’Ajuts Universitaris i de Recerca (AGAUR), Generalitat de Catalunya, 2017-SGR-01581, 08001 Barcelona, Spain; ivila@clinic.cat; 7 Otorhinolaryngology Head-Neck Surgery Department, Hospital Clínic, IDIBAPS Universitat de Barcelona, Villarroel 170, 08036 Barcelona, Spain; 8 Genómica Translacional y Terapias Dirigidas en Tumores Sólidos, IDIBAPS, 08036 Barcelona, Spain; 9 Departament de Cirurgia I Especialitats Mèdicoquirúrgiques, Facultat de Medicina, Universitat de Barcelona, 08036 Barcelona, Spain; 10 MoBioFood Research Group, Biochemistry and Biotechnology Department, Universitat Rovira i Virgili, 43002 Tarragona, Spain; ximena.terra@urv.cat; 11 Department of Biochemistry and Molecular Biology, Faculty of Pharmacy, Complutense University of Madrid, 28040 Madrid, Spain; pbragado@ucm.es; 12 Tissue Repair and Regeneration Laboratory, Department of Biosciences, Faculty of Sciences and Technology, University of Vic, 08500 Vic, Spain; gemma.fuster@uvic.cat; 13 Department of Biochemistry and Molecular Biomedicine, Institute of Biomedicine University of Barcelona (IBUB), 08028 Barcelona, Spain; 14 Otorhinolaryngology Department, Hospital de la Santa Creu i Sant Pau, Universitat Autònoma de Barcelona, 08193 Barcelona, Spain; 15 Centro de Investigación Biomédica en Red de Bioingeniería, Biomateriales y Nanomedicina (CIBER-BBN), 28001 Madrid, Spain; 16 Genomics of Complex Diseases, Research Institute Hospital Sant Pau, IIB Sant Pau, 08041 Barcelona, Spain; mcamacho@santpau.cat

**Keywords:** semaphorin-3F, neuropilin-2, occult lymph node metastases, elective neck dissection, head and neck squamous cell carcinoma, HNSCC, lymphangiogenesis

## Abstract

**Simple Summary:**

The presence of lymph neck node metastasis is the most important clinical prognostic factor in patients with head and neck squamous cell carcinoma. The expression of the semaphorin-3F and neuropilin-2 proteins is involved in the regulation of lymphangiogenesis. We analized the transcriptional expression of semaphorin-3F and neuropilin-2 in head and neck squamous cell carcinoma tumors of patients without clinical or radiology evidence of lymph node involvement treated with elective neck dissection. We found that patients with high semaphorin-3F and low neuropilin-2 had a lower risk of occult lymph node metastasis than the ones who had low semaphorin-3F or high semaphorin-3F and high neuropilin-2. If our results are validated, the expression of the SEMA3F-NRP2 axis could be used as a biomarker predicting the risk of occult lymph node metastasis, with elective neck dissections being indicated exclusively for patients at higher risk. The availability of a biomarker with the capacity to predict the presence of occult lymph node metastases would make it possible to avoid elective neck dissections in low-risk patients, with the consequent decrease in surgical time and morbidity of the treatment without impairing the oncologic outcome.

**Abstract:**

The expression of the semaphorin-3F (SEMA3F) and neuropilin-2 (NRP2) is involved in the regulation of lymphangiogenesis. The present study analyzes the relationship between the transcriptional expression of the SEMA3F-NRP2 genes and the presence of occult lymph node metastases in patients with cN0 head and neck squamous cell carcinomas. We analyzed the transcriptional expression of SEMA3F and NRP2 in a cohort of 53 patients with cN0 squamous cell carcinoma treated with an elective neck dissection. Occult lymph node metastases were found in 37.7% of the patients. Patients with occult lymph node metastases (cN0/pN+) had significantly lower SEMA3F expression values than patients without lymph node involvement (cN0/pN0). Considering the expression of the SEMA3F-NRP2 genes, patients were classified into two groups according to the risk of occult nodal metastasis: Group 1 (*n* = 34), high SEMA3F/low NRP2 expression, with a low risk of occult nodal involvement (14.7% cN0/pN+); Group 2 (*n* = 19), low SEMA3F or high SEMA3F/high NRP2 expression, with a high risk of occult nodal involvement (78.9% cN0/pN+). Multivariate analysis showed that patients in Group 2 had a 26.2 higher risk of lymph node involvement than patients in Group 1. There was a significant relationship between the transcriptional expression values of the SEMA3F-NRP2 genes and the risk of occult nodal metastases.

## 1. Introduction

Head and neck squamous cell carcinoma (HNSCC) is the seventh most common cancer type by incidence and mortality, with 890,000 new cases and 450,000 deaths worldwide in 2018 [1]. The presence of lymph neck node metastasis is the most important clinical prognostic factor in HNSCC patients.

Depending on the location and extent of the primary tumor, a variable proportion of patients with head and neck squamous cell carcinoma (HNSCC) without clinical or radiological evidence of lymph node involvement at diagnosis (cN0) have occult lymph node metastases (cN0/pN+) [2,3]. The presence of occult lymph node metastases justifies elective neck dissections in these patients and has a significant impact on survival [4].

The availability of biomarkers with the ability to predict the presence of occult lymph node metastases would make it possible to select patients who are candidates for elective treatment of the lymph node areas, limiting the intensity of treatment at the regional level in low-risk patients, with the consequent reduction in morbidity and impairment of quality of life.

Tumor lymphangiogenesis is a complex process involving interactions between endothelial cells, tumor cells, and the tumor microenvironment. One of the factors with the highest capacity to promote lymphangiogenesis is the vascular endothelial growth factor-C (VEGF-C), which appears overexpressed in many carcinoma models, correlating with the tumor lymphangiogenesis and the appearance of lymph node metastasis [5].

One of the receptors targeted by VEGF-C is neuropilin-2 (NRP2), which has been implicated in the process of VEGF-C-mediated lymphangiogenesis [6]. NRP2 was first identified as a receptor for semaphorin-3F (SEMA3F). Semaphorins are a family of proteins initially described in relation to processes of neuronal orientation. Semaphorins are involved also in the regulation of the angiogenesis process. Specifically, it has been described that SEMA3F could inhibit tumor growth and angiogenesis after binding with NRP2 in diverse human cell types [7,8].

Several studies have shown that the expression of SEMA3F and its receptors fulfill important regulatory roles in multiple forms of cancer [9], and that the use of treatments aimed at modifying the activity of the SEMA3F-NRP2 axis would have therapeutic potential [10,11].

High immunohistochemical expression of NRP2 in patients with HNSCC has been observed to increase lymphatic vascular density and lymphovascular invasion, with a higher risk of regional involvement and decreased survival [12,13]. Conversely, high expression of SEMA3F has been associated with a reduction in the degree of tumor lymphovascular infiltration and increased survival [12,14].

Considering these regulatory roles played by the SEMA3F-NRP2 axis in the lymphovascular infiltration process and lymph node involvement, we hypothesized that there is a relationship between the expression of these genes and the risk of occult lymph node metastasis in patients with HNSCC. The aim of the present study is to analyze the predictive capacity of the transcriptional expression of SEMA3F and NRP2 in determining the presence of occult lymph node metastases in patients with HNSCC.

## 2. Materials and Methods

### 2.1. Patients

With the exception of patients with a glottic cT1N0 tumor, our center’s treatment protocol includes the use of an elective neck dissection for all patients with HNSCC without clinical or radiological evidence of lymph node involvement at the time of diagnosis (cN0) and who received a surgical treatment of the primary location of the tumor.

The current study was carried out retrospectively by analyzing biopsies obtained from the primary location of the tumor prior to any treatment from patients with a pathologically confirmed squamous cell carcinoma located in the oral cavity, larynx or hypopharynx, without clinical or radiological evidence of lymph node involvement at the time of diagnosis (cN0) and treated with an elective uni- or bilateral neck dissection. Fifty-three patients met the inclusion criteria and were the basis for the present study. The clinical information was obtained from a database that prospectively collects information of all patients with malignant head and neck tumors treated in our center since 1985 [15].

All patients included in the study were assessed by the Oncology Board of our institution, which determined the convenience of carrying out an elective neck dissection. Table 1 shows the characteristics of the patients included in the study.

All the patients were treated with surgical resection of the primary location of the tumor combined with an elective uni- (*n* = 15, 28.3%) or bilateral (*n* = 38, 71.7%) neck dissection. In 31 patients the surgery was followed by adjuvant treatment with radiotherapy (*n* = 21) or chemoradiotherapy (*n* = 10). The criteria for adjuvant treatment were pT4 tumor extension, positive or close resection margins, three or more metastatic lymph nodes, or the presence of lymph node metastases with extracapsular spread. Given the interaction between tobacco and alcohol consumption, a combined variable of toxic consumption was created with three categories: no consumption; moderate consumption (<20 cigarettes/day and/or <80 g alcohol/day); and severe consumption (≥20 cigarettes/day or ≥80 g alcohol/day).

Based on the analysis of pathologic findings, we classified the patients according to the absence (cN0/pN0) or presence (cN0/pN+) of occult neck node metastases. For cN0/pN+ patients, we considered the uni- or bilateral involvement, as well as the presence of metastatic nodes with extracapsular spread. The mean number of dissected nodes per patient was 34.0 (SD = 17.3, range = 10–84).

The design of the study was approved by the Institutional Review Board of our center (IIBSP-CCC-14-93) and was performed in accordance with the principles outlined in the Declaration of Helsinki.

### 2.2. Transcriptional Analysis

Samples obtained from each patient were immediately suspended in RNA-later (Quiagen GmbH, Hilden, Germany) to prevent mRNA degradation, and stored at −80 °C until processing. Total RNA was extracted using Trizol (Invitrogen, Carlsbad, CA, USA) according to the manufacturer’s instructions. The cDNA was obtained by reverse transcription of 1 g of RNA with High-Capacity cDNA Archive Kit (Applied Biosystems, Foster City, CA, USA) and transcriptional expression of NRP2, SEMA3F, and beta-actin as an endogenous control was assessed by RT-PCR on an ABI Prism 7000 using predesigned validated assays (TaqMan Gene Expression Assays; Applied Biosystems).

### 2.3. External Validation Sutyd: The Cancer Genome Atlas Database

The Cancer Genome Atlas (TCGA) [16] is a public data base that includes clinical information and the transcriptome of 528 patients with HNSCC and 43 samples of healthy mucosa. In order to obtain an external validation cohort, we conducted a study including 176 patients of the TCGA with a cN0 HNSCC for which information on the pathologic results of the elective neck dissection was available. Table S1 of the Supplementary Material shows the characteristics of the patients included in the validation study.

### 2.4. Statistical Analyses

The distribution of the transcriptional expression values of SEMA3F did not meet normality criteria (Kolmogorov–Smirnov test, *p* = 0.008), so the measures of central tendency were expressed using the median value, and nonparametric techniques were used in the comparison between the expression values. SEMA3F and NRP2 expression values were compared according to the location of the primary tumor, toxic consumption, histological grade, clinical category of local tumor extension (cT), and the presence of occult lymph node metastases (pN0 versus pN+). Comparisons were performed using the Mann–Whitney U test or the Kruskal–Wallis test depending on the application conditions. We proceeded to classify patients considering the presence of occult lymph node metastases as the dependent variable according to SEMA3F and NRP2 transcriptional expression values with a classification and regression tree (CRT) analysis. The hazard ratio for the presence of occult lymph node metastases was analyzed as a function of the categories obtained with the CRT with a logistic regression model. Disease-specific survival was estimated according to the categories obtained with the CRT analysis with the Kaplan–Meier method, using the log-rank test in the comparison of the survival curves. Finally, a multivariate analysis (logistic regression) was performed considering the presence of occult lymph node metastases as the dependent variable and including in the model the location of the primary tumor, the local extension of the tumor (cT), and the categories obtained with the CRT. Comparison between the transcriptional expression of SEMA3F and NRP2 between normal and tumor mucosa samples from patients included in TCGA was performed with a paired sample t-test. Statistical analysis was performed with the SPSS 26.0.0.1 software.

## 3. Results

Twenty patients included in the study (37.7%) presented occult lymph node metastases (cN0/pN+) in the elective neck dissections, 17 unilaterally and 3 bilaterally. Of the cN0/pN+ patients, in three cases one of the positive lymph nodes presented extracapsular spread.

### 3.1. SEMA3F/NRP2 Expression According to Clinical Variables

When analyzing the levels of NRP2 transcriptional expression depending on the clinical variables, no significant differences appeared according to the location of the primary tumor (*p* = 0.449), toxics consumption (*p* = 0.284), category of local extension of the tumor (*p* = 0.255), or histological grade (*p* = 0.921). Similarly, no significant differences appeared in the SEMA3F transcriptional expression according to the primary tumor location (*p* = 0.394), toxic consumption (*p* = 0.770), local extension of the tumor (*p* = 0.794), or histological grade (*p* = 0.647).

There were no significant differences in NRP2 transcriptional expression according to the presence of occult lymph node metastases (*p* = 0.545), whereas patients with occult lymph node metastases (cN0/pN+) had significantly lower SEMA3F expression values than patients without lymph node involvement (cN0/pN0) (*p* = 0.006). Figure S1 of the Supplementary Material shows the distribution of NRP2 and SEMA3F values as a function of the pathological status of the neck dissections. Table S2 of the Supplementary Material shows the median NRP2 and SEMA3F expression values as a function of the clinical variables analyzed.

### 3.2. Classification Depending on the SEMA3F/NRP2 Expression

**Figure 1:**
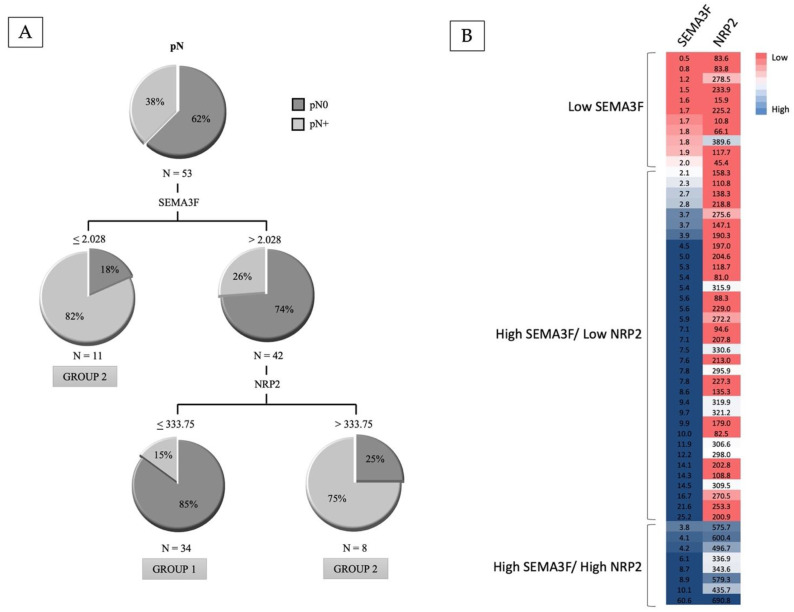
(**A**): Classification tree according to the transcriptional expression values of SEMA3F and NRP2 considering the presence of occult lymph node metastases as the dependent variable. (**B**): Heatmap of SEMA3F and NRP2 according to the results obtained in the classification tree.

Considering the presence of occult lymph node metastases as the dependent variable, the CRT classified the patients into three categories, with a first partition according to SEMA3F expression values, and a second partition level that only affected cases with high SEMA3F expression depending on the NRP2 expression values (Figure 1A). Figure 1B shows the heatmap of SEMA3F and NRP2 according to the results obtained in the classification tree. Three terminal nodes were obtained: patients with low SEMA3F expression (*n* = 11, 20.8%; 81.8% pN+); patients with high SEMA3F/high NRP2 expression (*n* = 8, 15.1%; 75% pN+); and patients with high SEMA3F/low NRP2 expression (*n* = 34, 64.2%; 14.7% pN+). The two terminal nodes with a high risk of occult lymph nodal metastases were grouped, classifying patients into two groups: Group 1, high SEMA3F/low NRP2 expression (*n* = 34), with a low risk of occult nodal involvement (14.7% cN0/pN+); Group 2, low SEMA3F expression or high SEMA3F/high NRP2 expression (*n* = 19), with a high risk of occult nodal involvement (78.9% cN0/pN+). Relative to Group 1, the hazard ratio of the presence of occult lymph node metastases for patients included in Group 2 was 21.7 (95% CI, HR = 5.0–93.1).

According to the result of the multivariate analysis (Table 2), the only variable that was significantly related to the risk of occult lymph node metastasis was the classification obtained from the SEMA3F-NRP2 expression values. Relative to patients with a low risk of occult nodal involvement (Group 1), patients included in Group 2 had a 26.2-fold increased risk of nodal involvement (95% CI 5.1–132.4, *p* < 0.001). Neither the location of the primary tumor nor the local extension of the tumor was related to the risk of occult nodal involvement.

The transcriptional profile of the SEMA3F-NRP2 genes was reflected in the patients’ disease-free survival. Five-year disease-free survival rate of patients included in Group 1 (high SEMA3F/low NRP2 expression, *n* = 34) was 74.6% (95% CI 57.6–91.6%), significantly higher than that of those patients included in Group 2 (low SEMA3F or high SEMA3F/high NRP2 expression, *n* = 19), whose rate was 27.1% (95% CI 3.6–50.6%) (*p* = 0.0001). Figure S2 of the Supplementary Material shows the disease-free survival curves according to the SEMA3F-NRP2 transcriptional profile.

### 3.3. Results of the External Validation Study with the TCGA Data

In the external validation study, occult lymph node metastases were found in 56 (31.8%) of the 176 patients of the TCGA. The CRT analysis classified the patients into three groups considering the transcriptional expression of the SEMA3F-NRP2 genes. A first partition was obtained according to the SEMA3F expression values, and a second partition only affected cases with low SEMA3F expression depending on the NRP2 expression values. Figure S3 of the Supplementary Material shows the resulting classification tree. Patients with high SEMA3F expression (*n* = 46) had a low risk of occult lymph node metastases (15.2%). Patients with low SEMA3F/low NRP2 expression (*n* = 72) had an intermediate risk of occult lymph node metastases (30.6%). Finally, patients with low SEMA3F/high NRP2 expression (*n* = 58) were the group that had the highest risk of occult lymph node metastases (46.6%).

We compared the transcriptional expression of the tumor and the healthy mucosa of the patients of the TCGA. The expression of SEMA3F was higher in healthy mucosa than in the tumor (*p* = 0.015) and, in contrast, the expression of NRP2 was higher in the tumor than in healthy mucosa (*p* = 0.001). Figure S4 of the Supplementary Material shows the distribution of the transcriptional expression values of SEMA3F and NRP2 in the samples of healthy mucosa and tumor.

## 4. Discussion

According to our results, the transcriptional expression of the SEMA3F-NRP2 genes was significantly associated with the risk of occult lymph node metastases in neck dissections carried out electively in patients with HNSCC. The result of multivariate analysis showed that tumors with low SEMA3F expression or high SEMA3F expression associated with high NRP2 expression had a more than 25-fold higher risk of occult lymph node metastasis than tumors with high SEMA3F and low NRP2 expression.

The SEMA3F gene is located on 3p21, a frequently deleted loci in HNSCC, being one of the most commonly under-expressed genes [17]. Immunohistochemical and transcriptional studies in patients with HNSCC have shown a reduction in the expression of SEMA3F relative to healthy mucosa [14,18]. In agreement with these findings, when analyzing the results included in TCGA we could observe that the normal mucosal samples had higher transcriptional expression values than those observed in the tumor sample, while NRP2 expression behaved in the opposite way, with a higher expression in the tumor samples. All these findings lead us to suppose that the reduction in the expression of SEMA3F and the increase in NRP2 should be considered as elements that favor the carcinogenesis process. Moreover, in vitro studies have found an increase in cell proliferation, migration, invasion capacity, and higher vascular recruitment when SEMA3F expression was suppressed, thus facilitating the angiogenesis process through its interaction with NRP2. Additionally, in vivo studies established that the induction of SEMA3F expression resulted in the inhibition of the tumor growth, as well as a significant reduction in tumor lymphangiogenesis and lymph node involvement [14,18]. Zhang et al. [12] found a relationship between the decrease in SEMA3F expression and an increase in the risk of regional involvement with a significant decrease in survival in patients with oral squamous cell carcinomas. Similarly, Xie et al. [19] established an association between the loss of SEMA3F expression and a significant increase in the risk of lymph node involvement in patients with esophageal squamous cell carcinoma, a tumor model that shares many characteristics with HNSCC.

In patients with cN0 oral cavity carcinomas, Ong et al. evidenced a direct relationship between NRP2 immunopositivity and regional involvement, with high NRP2 expression being associated with a significant reduction in survival [13]. The inhibition of NRP2 expression induced a significant reduction in proliferation, migration, and invasion capacity. In the same way, Zhang et al. also described a significant correlation between a high NRP2 expression at the oral tumor primary location and a high lymphatic density and lymphatic vascular invasion, entailing an increased frequency of lymph node involvement and a significant decrease in survival [12].

The increased risk of lymph node involvement in patients with reduced SEMA3F expression seems to be related to the loss of the ability to directly suppress the lymphangiogenesis process as well as the decrease in NRP2 receptor occupation, facilitating the competitive activity of other specific ligands with the capacity to promote lymph node metastasis such as VEGF-C [12,14]. On the other hand, even in the presence of SEMA3F, the high expression of NRP2 would allow its activity as a potent promoter of tumor-associated lymphangiogenesis [13]. However, we did not conduct mechanistic studies to demonstrate these changes in enzyme activity or its downstream effects, nor its potential relationship with VEGF-C. Although the predictive ability of the SEMA3F/NRP2 transcriptional expression is clearly demonstrated in our study, the involvement of this pathway in the progression of HNSCC requires further study.

In addition to the increased risk of lymphatic metastasis, high NRP2 and low SEMA3F expression has been associated with an increase in tumor aggressiveness, evidenced as an increase in the capacity for proliferation and infiltration [12,13,14,18]. Taken together, these findings support the significant relationship between the pattern in the transcriptional expression of the SEMA3F-NRP and the patient survival observed in our study.

Comparing the results obtained with the patients of the TCGA with our sample, we can observe that predictive capacity of the transcriptional expression of the SEMA3F/NRP2 genes in detecting the presence of occult lymph node metastases was maintained. Both expressions of low SEMA3F and a high NRP2 were associated with an increased risk of occult lymph node metastases.

According to the results obtained in various meta-analyses [20,21] and clinical trials [22], elective neck dissections can significantly reduce the rate of regional nodal recurrence and improve the disease-free survival in patients with HNSCC without clinical or radiologic evidence of lymph node metastasis in the neck (cN0). However, these neck dissections involve carrying out neck surgery in a proportion of patients who will not obtain therapeutic benefit from the procedure. One of the approaches to minimize the proportion of patients who do not benefit from the elective neck dissection is the sentinel lymph node biopsy. Another strategy is to have biomarkers with the capacity of predicting the risk of occult lymph node metastases in cN0 patients.

If our results are validated, the expression of the SEMA3F-NRP2 axis could be used as a biomarker predicting the risk of occult lymph node metastasis, with elective neck dissections being indicated exclusively for patients at higher risk. Patients with a low risk could be candidates for observation or sentinel node biopsy. The availability of a biomarker with the capacity to predict the presence of occult lymph node metastases would make it possible to avoid elective neck dissections in low-risk patients, with the consequent decrease in surgical time and morbidity of the treatment without impairing the oncologic outcome.

Several authors have studied the relationship between the expression of molecular biomarkers and the risk of occult lymph node metastases in patients with HNSCC. Table 3 shows a summary of the studies performed. Most of the studies have analyzed the different markers by determining immunohistochemical expression, mainly in patients with tumors located in the oral cavity, obtaining variable results in terms of prognostic capacity. The results of our study are among those that achieved the highest accuracy rate (83% of cases correctly classified), with a sensitivity of 75% and relatively high specificity and negative predictive values (87.7% and 85.2%, respectively).

Moreover, the presence of occult lymph node metastases has been associated with certain histological features of the tumor such as the degree of lymphatic invasion [34], an aggressive pattern of the tumor infiltration margin [35], the depth of infiltration [22,34,36], the presence of perineural invasion [35], the presence of myofibroblasts in the tumor stroma [37], the eosinophilic infiltration of the tumor [38], or the appearance of hybrid circulating cells [39].

Further studies evaluating in conjunction the predictive capacity of two or more of these molecular and/or histological markers are needed in order to find a combination of biomarkers with sufficient sensitivity and specificity to safely select cN0 patients who are not candidates for elective neck dissection.

The present study has limitations inherent to its retrospective nature and a small sample of patients. Future prospective external validation studies in large cohorts of patients are needed before we can consider that the transcriptional activity of the SEMA3F-NRP2 genes has sufficient predictive capacity to avoid elective neck dissections in patients with HNSCC.

## 5. Conclusions

According to the results obtained in our study, there is a significant relationship between the transcriptional expression of the SEMA3F-NRP2 genes and the risk of occult lymph node metastasis in patients with HNSCC. The risk of occult lymph node metastasis was low for the group of patients with high SEMA3F/low NRP2 expression, while it was high for patients with low SEMA3F or high SEMA3F/high NRP2 expression.

## Figures and Tables

**Table 1:** Characteristics of the patients included in the study.

Characteristics		*N* Patients (%)
Age	Mean 66.7 years/Range 31.1–87.3 years	
Gender	Men	44 (83.0%)
	Women	9 (17.0%)
Location	Oral cavity	15 (28.3%)
	Hypopharynx	3 (5.7%)
	Larynx	35 (66.0%)
Toxic consumption	No	7 (13.2%)
	Moderate	13 (24.5%)
	Severe	33 (62.3%)
Local extension	cT1–2	4 (7.5%)
	cT3	22 (41.5%)
	cT4	27 (50.9%)
Histologic grade	Well differentiated	3 (5.7%)
	Moderately differentiated	45 (84.9%)
	Poorly differentiated	5 (9.4%)
Adjuvant treatment	No	22 (41.5%)
	Radiotherapy	21 (39.6%)
	Chemoradiotherapy	10 (18.9%)

**Table 2:** Results of a multivariate analysis considering the appearance of occult lymph node metastases as the dependent variable (HR: hazard ratio).

Variation		HR	CI 95% HR	*p*
Location	Oral cavity	1		
	Hypopharynx	0.34	0.010–12.30	0.558
	Larynx	0.25	0.04–1.44	0.121
Local extension	cT1-2	1		
	cT3	0.14	0.06–3.41	0.233
	cT4	0.18	0.08–4.14	0.286
SEMA3F-NRP2	Group 1	1		
	Group 2	26.21	5.19–132.42	0.000

**Table 3:** Results obtained in studies that have analyzed the relationship of molecular biomarkers with the presence of occult lymph node metastases in patients with HNSCC (S, sensitivity; E, specificity; PPV, positive predictive value; NPV, negative predictive value; AI, accuracy index; OC, oral cavity; ORF, oropharynx; HNSCC, head and neck squamous cell carcinoma; IHC, immuno-histochemistry). * Numerical aberrations of CCND1; ** Serum MYO5A levels.

Author (Year)	Biomarker	Location	*n*	% pN+	Determination	Related to	S	E	PPV	NPV	AI
Franchi (1996) [23]	PCNA	Larynx	60	50%	IHQ	pN+	80.0%	80.0%	80.0%	80.0%	80.0%
	MIB-1					pN+	56.6%	83.3%	77.2%	65.7%	70.0%
	E-cadherin					pN0	50.0%	86.6%	78.9%	63.4%	68.3%
Capaccio (2000) [24]	Cyclin D1	HNSCC	96	33.3%	IHQ	pN+	68.7%	68.7%	52.3%	81.4%	68.7%
Myo (2005) [25]	Cyclin D1 *	OC	45	37.7%	FISH	pN+	70.5%	89.2%	80.0%	83.3%	82.2%
Huber (2011) [26]	E-cadherin	OC, ORF	120	37.5%	IHQ	pN0	82.2%	44.0%	46.8%	80.4%	58.3%
Zullig (2013) [27]	SOX2	OC	120	37.5%	IHQ	pN0	95.6%	32.0%	45.7%	92.3%	55.8%
Kelner (2014) [28]	Activin A	OC	110	26.35	IHQ	pN+	74.0%	56.4%	37.0%	86.2%	58.2%
Noorlag (2016) [29]	Cyclin D1	OC	152	25.0%	IHQ	pN+	63.1%	66.6%	38.7%	84.4%	65.7%
Mermod (2016) [30]	PROX1	OC, ORF	52	19.2%	IHQ	pN+	60.0%	98.0%	86.0%	91.0%	88.0%
Mermod (2018) [31]	CD31	OC, ORF	56	19.6%	IHQ	pN+	91.0%	65.0%	40.0%	97.0%	71.0%
Zhao (2018) [32]	MYO5A **	Larynx	103	31.0%	ELISA	pN+	77.8%	75.4%	-	-	-
Boeve (2021) [33]	Cortactin	OC	33	18.1%	IHQ	pN+	66.7%	88.8%	57.1%	92.3%	84.8%
Current study	SEMA3F	CECC	53	37.7%	PCR	pN0	75.0%	87.8%	78.9%	85.2%	83.0%
	NRP2					pN+					

## Data Availability

Nacional Cancer Institute. The Cancer Genome Atlas Program. Available online: https://tcga-data.nci.nih.gov/tcga (accessed on 22 February 2022).

## Supplementary Materials

The following are available online at https://www.mdpi.com/article/10.3390/cancers14092259/s1, Figure S1: Distribution of the transcriptional expression values of NRP2 and SEMA3F according to the pathologic status of the neck; Figure S2: Disease-free survival according to the expression categories of the SEMA3F-NRP2 genes; Figure S3: Classification tree according to the transcriptional expression values of SEMA3F and NRP2 of the patients included in The Cancer Genome Atlas considering the presence of occult lymph node metastases as the dependent variable; Figure S4: Distribution of the transcriptional expression values of NRP2 and SEMA3F in the samples of the healthy mucosa and the tumor of the patients included in The Cancer Genome Atlas; Table S1: Characteristics of the patients of The Cancer Genome Atlas included in the validation study; Table S2: Median of the transcriptional expression values of SEMA3F and NRP2 according to the characteristics of the patients included in the study.
