# Supplementary material for: Semaphorin-3F/Neuropilin-2 Transcriptional Expression as a Predictive Biomarker of Occult Lymph Node Metastases in HNSCC
Source: Cancers (Basel). 2022 Apr 30;14(9):2259. doi: 10.3390/cancers14092259 (PMC9100497; doi:10.3390/cancers14092259)
Supplement: Supplementary file 1 [file cancers-14-02259-s001.zip › cancers-1668612-supplementary.pdf]

Supplementary material

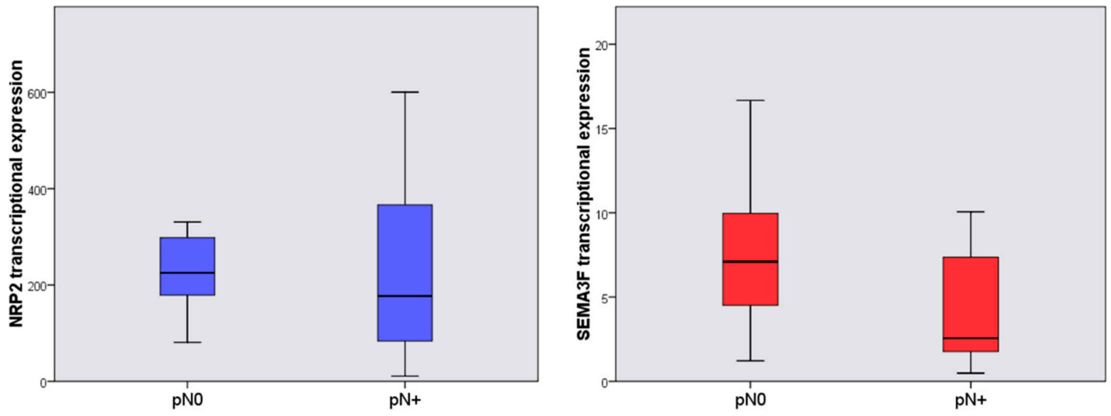

Figure S1. Distribution of the transcriptional expression values of NRP2 and SEMA3F according to the pathologic status of the neck.

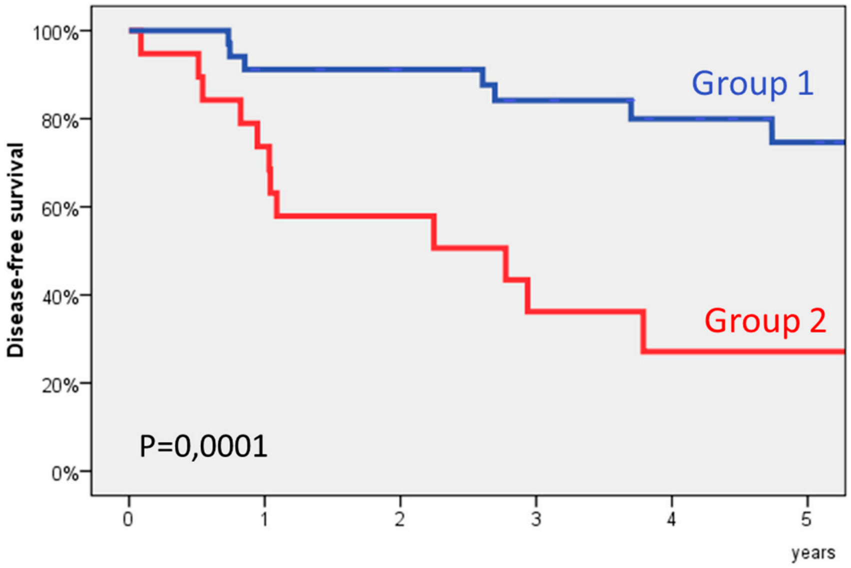

Figure S2. Disease-free survival according to the expression categories of the SEMA3F-NRP2 genes.

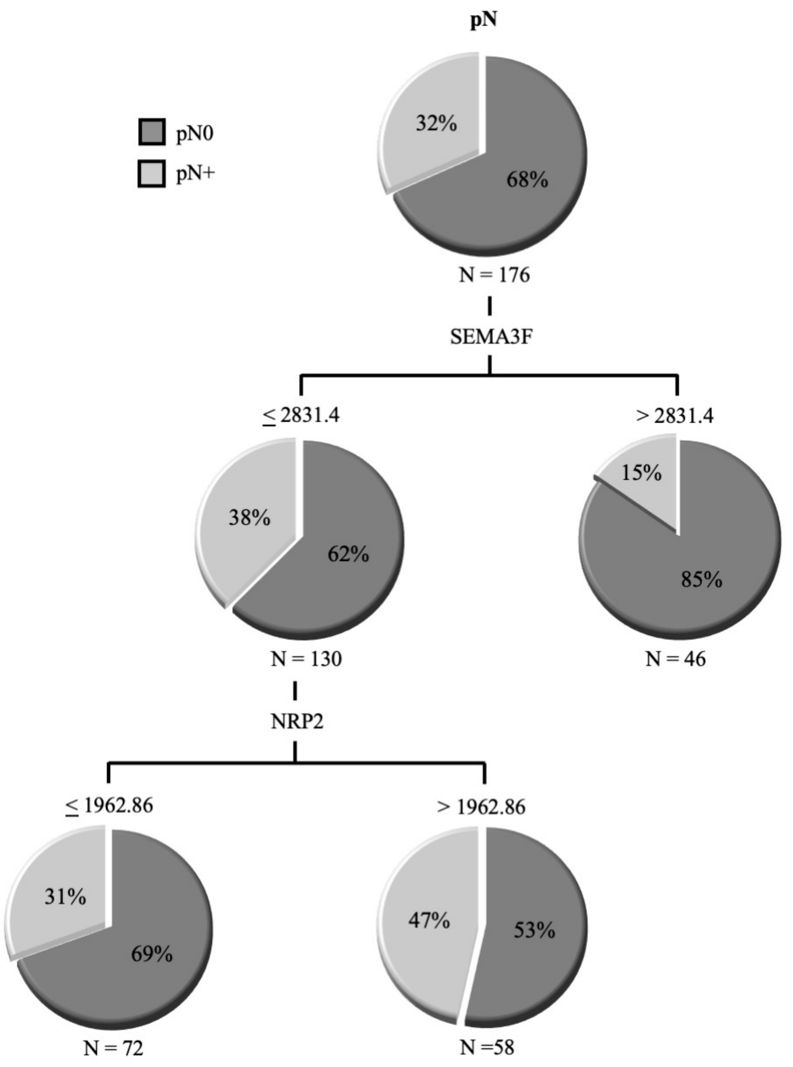

Figure S3. Classification tree according to the transcriptional expression values of SEMA3F and NRP2 of the patients included in The Cancer Genome Atlas considering the presence of occult lymph node metastases as the dependent variable.

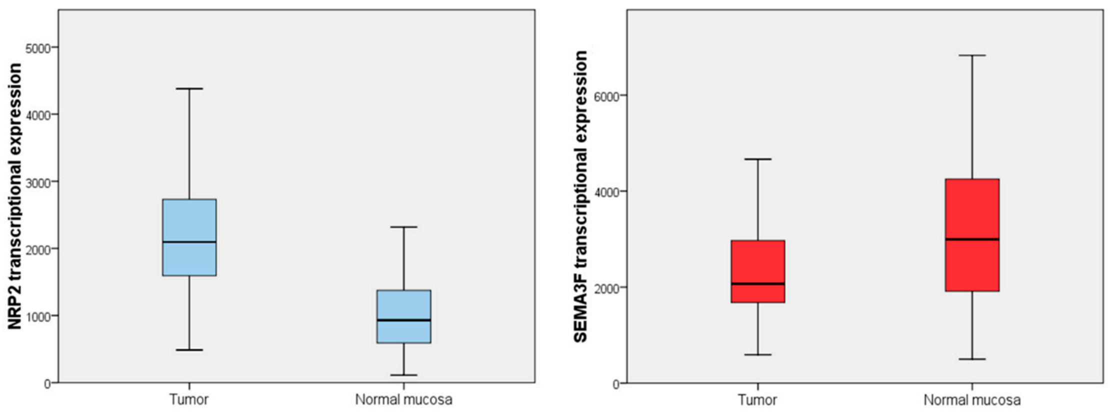

Figure S4. Distribution of the transcriptional expression values of NRP2 and SEMA3F in the samples of the healthy mucosa and the tumor of the patients included The Cancer Genome Atlas.

Table S1. Characteristics of the patients of The Cancer Genome Atlas included in the validation study.

		N (%)
**Mean age (SD) years**		63.1 (12.0)
**Gender**	Male	128 (72.7%)
	Female	48 (27.3%)
**Location**	Oral cavity	123 (69.9%)
	Oropharynx	11(6.3%)
	Hypopharynx	1 (0.6%)
	Larynx	41 (23.3%)
**Clinical local extension**	cT1	16 (9.1%)
	cT2	55 (31.3%)
	cT3	45 (25.6%)
	cT4	60 (34.1%)

Table S2. Median of the transcriptional expression values of SEMA3F and NRP2 according to the characteristics of the patients included in the study.

		SEMA3F	*p*	NRP2	*p*
**Location**	Oral cavity	4.51	0.394	233.8	0.449
	Hypopharynx	7.60		309.5	
	Larynx	5.57		202.8	
**Toxic consumption**	No	3.78	0.770	218.8	0.284
	Moderate	5.32		270.5	
	Severe	5.85		200.9	
**Local extension**	cT1-2	4.63	0.794	350.6	0.255
	cT3	6.47		248.6	
	cT4	5.61		202.8	
**Regional extension**	pN0	7.09	0.006	225.2	0.545
	pN+	2.55		177.0	
**Histologic grade**	Well differentiated	4.51	0.647	270.5	0.921
	Moderately differentiated	5.85		213.0	
	Poorly differentiated	5.02		225.2	
